# Changes in health-related quality of life and clinical implications in Chinese patients with chronic cough

**DOI:** 10.1186/1745-9974-5-7

**Published:** 2009-09-25

**Authors:** Wei Ma, Li Yu, Yu Wang, Xin Li, Hanjing LÜ, Zhongmin Qiu

**Affiliations:** 1 Department of Respiratory Medicine, Tongji Hospital, School of Medicine, Tongji University, No.389 Xincun Road, Shanghai 200065, PR China

## Abstract

**Background:**

Chronic cough has negative effects on quality of life. However, the changes in health-related quality of life and clinical implications remain unclear in Chinese patients with chronic cough.

**Methods:**

A standard Chinese version of Leicester cough questionnaire (LCQ) was developed by an established translation procedure and its repeatability was assessed in a preliminary study involving 20 untreated patients with stable chronic cough. The quality of life was measured with the Short form-36 health survey and compared between 110 patients with chronic cough and 90 healthy volunteers. The changes in health-related quality of life were evaluated in the patients with chronic cough with the LCQ just before the specific treatment was initiated and a week after the cough had resolved completely. Cough threshold with inhaled capsaicin, expressed as the lowest concentration of capsaicin required for the induction of ≥5 coughs, was also measured.

**Results:**

The repeatability of the Chinese version of the LCQ was validated at a four day interval with the intraclass correlation coefficients of 0.89-0.94 for total and domain score (n = 20). The scores of the Short form-36 health survey were significantly lower in patients with chronic cough than those in healthy volunteers. In general, there was no significant difference in overall quality of life between different causes of chronic cough or genders although embarrassment, frustration and sleep disturbance were more common in female patients, as indicated by the LCQ. However, the successful treatment of cough obviously increased the total scores of the LCQ from 14.2 ± 2.7 to 19.5 ± 1.9 (t = 13.7, *P *< 0.0001). There was a significant correlation between the total score of the LCQ and physical (r = 0.39, *P *< 0.0001) or mental (r = 0.30, *P *< 0.001) component summary of the Short form-36 health survey but not between the LCQ and capsaicin cough threshold.

**Conclusion:**

The quality of life is significantly impaired in Chinese patients with chronic cough. The Chinese version of the LCQ is a valid measure of cough related quality of life and is repeatable and responsive.

## Background

Chronic cough is a common symptom that involves 20%-38% of the patients seeking medical advice in the respiratory clinic [1,2], and a medical problem often faced by clinicians. Persistent cough may cause organ injuries as well as psychological and social dysfunction, thereby having a profound adverse impact on quality of life [3]. With quantitative or semi-quantitative methods, the changes in the health-related quality of life (HRQOL) provoked by cough can be accurately analyzed, which is useful for assessment of cough severity and therapeutic efficacy, and can help to guide clinical practice and research in chronic cough.

Cough symptom score, generic instruments such as the Short form-36 health survey (SF-36), Sickness impact profile and the other respiratory health questionnaires were common tools for the evaluation of HRQOL in chronic cough in early years [4]. Cough symptom score, although simple and convenient, is not comprehensive while generic instruments are troublesome and time-consuming. Furthermore, lack of scores specific for cough or only several items referring to cough in generic instruments makes it difficult to precisely measure the tiny changes of HRQOL caused by cough. To overcome these shortages, the tools specific for assessments of HRQOL on chronic cough have been designed, including the cough-specific quality-of-life questionnaire [5], Leicester cough questionnaire (LCQ) [6] and chronic cough impact questionnaire [7]. The utilization of these instruments has greatly promoted the studies of quality of life in chronic cough patients.

It has been demonstrated that chronic cough has negative effects on HRQOL of patients in many aspects including physical, psychological and social domains, among which the adverse impact on the psychosocial domain is the most notable and possibly related to gender [5]. Successful treatments not only relieve the symptoms of cough, but also improve the quality of life [5,7,8]. However, the current data on the HRQOL of chronic cough are only from several western countries. The conclusions extrapolated from these studies might not be suitable for the patients in the other regions of the world because of differences in the geography, ethnic, customs, cultural backgrounds and lifestyle. Therefore, the purpose of the present study was to investigate the changes in HRQOL in Chinese patients with chronic cough.

## Methods

### Subjects

110 patients with chronic cough referred consecutively to the Department of Respiratory Medicine in Tongji hospital were selected for the study. They were eligible for inclusion in case of the presence of an isolated persistent cough of > 8 weeks, the absence of adventitious lung sounds on physical examination, normal findings on plain chest radiography, a forced expiratory volume in 1 s (FEV_1_) > 80% of predicted and a ratio with forced vital capacity (FEV_1_/FVC) >70%. Current smokers and ex-smokers of < 2 years were excluded.

90 healthy volunteers were enrolled from the staff and medical students in the hospital. All of them were lifetime non-smokers, had no history of chronic respiratory disease or allergic disease, had no upper respiratory tract infections in the previous 2 months and did not currently cough.

The general data of patients with chronic cough and healthy volunteers were shown in Table 1. There was no significant difference in age, the distribution of gender and the variables of lung function between the two groups. The hospital institutional ethics committee approved the study, and all subjects gave informed consent. The partial results of this study have been published in a form of meeting abstract [9].

**Table 1:** General data of chronic cough group and healthy control group

**Items**	**Chronic cough group**	**Healthy volunteers**
Cases	110	90
Male/female	36/74	40/50
Ages (yr)	47 ± 14	47 ± 12
Cough duration (month, range)	9(2-360)	0
FEV_1_(% predictive value)	95 ± 15	98 ± 12
FVC (% predictive value)	123 ± 16	120 ± 18
FEV_1_/FVC (%)	81 ± 7	83 ± 9

FEV1: forced expiratory volume in one second; FVC: forced vital capacity.

### Methods and measurements

#### 1. SF-36

A validated Chinese version of SF-36 [10] was employed. The questionnaire is self-administered and composed of 8 multi-item scales (36 items) assessing physical function (PF), role-limitations due to physical problems (RP), social function (SF), bodily pain (BP), role-limitations due to emotional problems (RE), mental health (MH), general health (GH) and vitality (VT). Generally, it took less than 5 minutes for patients to complete. For each multi-item scale, item scores are coded, summed and transformed on to a scale from 0 (worst health) to 100 (best health) [11], then got a standard score of 0-100 [12]. Among them, PF, RF, BP and GH make up the physiological domain, and these 4 items derive the physical component summary (PCS). In contrast, RE, VT, MH and SF comprise the psychosocial domain and derive the mental component summary (MCS) [13]. The higher score means better quality of life.

#### 2. LCQ

After obtaining the written permission from the designer of LCQ, Dr Birring, we translated it to standard Chinese. The translation followed an established forward-backward translation procedure, with independent translations and counter-translation. Independent translations into Chinese of LCQ by two doctors specialized in respiratory medicine were pooled to a common version. A psychiatric doctor fluent in English translated this provisional Chinese version back into English. This back translation was found to be almost identical to the source document. The Chinese version of LCQ was then finally determined [see Additional file 1]. LCQ consists of 19 items, involving physical, psychological and social domains, and each item represents an adverse event caused by cough [6]. A 7-point Likert scale is used for scoring the answer. Like SF-36, LCQ is also designed for self-administration and needs less than 5 minutes for its completion, a higher score reflects better health status.

A preliminary study was performed to validate the repeatability (test-retest reliability) of the Chinese version of LCQ over time. The reliability was assessed by administering the LCQ to 20 untreated patients with stable chronic cough at baseline and four day later when the patients did not feel that their cough had changed.

#### 3. Cough sensitivity test with capsaicin

cough sensitivity was measured according to the method described by Fujimura [14] with minor modifications. Briefly, the patient inhaled an aerosolized control solution of physiological saline followed by progressively increasing double concentrations (0.49-1000 μmol/L) of the capsaicin solution (Wako Pure Chemical Ind., Japan), delivered through a PARI BOY N085 air-compressed nebulizer (PARI GmbH, German) at an output rate of 0.5 ml/min with a mass median diameter of the particles in 3.7 μm. Each concentration of solution was inhaled by tidal mouth-breathing for 15 s, the number of cough was counted from the initiation of a 15 s inhalation of capsaicin solution to a subsequent 45 s observation time. The cough threshold was defined as the lowest concentration of capsaicin required for the induction of ≥5 coughs (C5). When the maximal concentration of capsaicin was attained and the subjects had no cough, 1000 μmol/L was assumed as a value of C5.

### Study design and follow up

The etiologies of chronic cough were identified and treated by our established protocol [15]. Just before the specific treatment was initiated, SF-36 and LCQ were filled out by the patients under the guidance of doctors and capsaicin cough threshold C5 was detected. HRQOL assessed with the LCQ was re-evaluated one week after the patients reported that their cough had completely resolved with successful treatment specific for the etiology of the chronic cough.

### Statistical analysis

The data was expressed as  ± *s *except for duration of cough and cough threshold C5 which were presented as median (range). Comparisons of variables between the patients with chronic cough and healthy volunteers were made using unpaired *t*-tests while the difference of gender distribution was examined using *X*^2 ^analysis. Changes in HRQOL before and after treatment were analyzed by paired t tests. Comparisons between the different etiologies of chronic cough were performed with one way analysis of variance followed by *q*-test. The repeatability of Chinese version of LCQ was analyzed by calculating intraclass correlation coefficients of LCQ total and domain scores between the two evaluations. The relationships between log transformed cough threshold C5 (Log C5) and LCQ or SF-36 score were calculated using Spearman rank order correlation coefficient. SPSS 10.0 software (SPSS Inc., Chicago, IL, USA) was used for statistical calculations. A P-value < 0.05 was considered significant.

## Results

### Validation of repeatability on the Chinese version of LCQ

The intraclass correlation coefficient of LCQ repeatability was 0.94 (95% confidence interval: 0.85-0.98, *P *= 0.000) for total score, 0.89 (95% confidence interval: 0.73-0.96, *P *= 0.000) for the physical domain score, 0.93 (95% confidence interval: 0.82-0.97, *P *= 0.000) for the psychological domain score and 0.92 (95% confidence interval: 0.79-0.97, *P *= 0.000) for the social domain score respectively.

### HRQOL in patients with chronic cough

HRQOL in 110 patients with chronic cough, as represented by scores of SF-36, was significantly poorer than that in 90 healthy volunteers. Among the multi-item scales of SF-36, RE, GH and RP were affected in the most outstanding way (Table 2).

**Table 2:** Comparison on scores of SF-36 between chronic cough group and healthy control group

**Items**	**Chronic cough group (n = 110)**	**Healthy volunteers (n = 90)**	**t value**	***P *value**
PF	88.9 ± 10.6	95.2 ± 6.7	5.1	0.000
RP	66.3 ± 34.5	92.0 ± 25.3	5.6	0.000
RE	44.5 ± 36.5	92.6 ± 21.0	11.6	0.000
VT	70.8 ± 16.6	86.7 ± 10.0	8.3	0.000
MH	75.8 ± 14.6	87.3 ± 9.8	6.7	0.001
SF	84.9 ± 16.7	93.9 ± 9.9	4.6	0.000
BP	89.5 ± 10.7	92.2 ± 10.1	1.9	0.063
GH	58.8 ± 18.4	83.8 ± 13.4	10.7	0.000
PCS	60.3 ± 5.0	66.2 ± 4.3	8.8	0.000
MCS	57.6 ± 5.6	66.0 ± 3.8	12.7	0.000

Abbreviation: PF, Physical function; RP, role-limitations due to physical health; SF, social function; BP, bodily pain; RE, role-limitations due to emotional problems; MH, mental health; GH, general health; VT, vitality; PCS, physical component summary; MCS, mental component summary

### Differences of HRQOL between different etiologies of chronic cough

The causes of chronic cough in 110 patients were shown in Table 3. Beside the single cause, cough symptoms in 10 patients could be explained by two causes, including 4 cases of cough variant asthma (CVA) plus upper airway cough syndrome (UACS), 4 cases of CVA plus gastroesophageal reflux disease (GERD) and 2 cases of UACS plus GERD. The other causes consisted of one case of environmental factor related cough, 3 cases of angiotensin-converting enzyme inhibitor-induced cough and 7 cases of idiopathic cough. There were no significant differences in HRQOL between the different etiologies of chronic cough, whether measured with LCQ (Table 3) or with SF-36 (Table 4).

**Table 3:** HRQOL comparison of LCQ scale between different causes of chronic cough (n = 110)

**Causes**	**Cases**	**Total score**	**Physical domain**	**Psychological domain**	**Social domain**
UACS	10	14.5 ± 0.7	4.6 ± 0.2	4.4 ± 0.3	5.5 ± 0.3
CVA	54	14.2 ± 0.4	4.6 ± 0.1	4.3 ± 0.1	5.3 ± 0.2
GERD	6	13.7 ± 0.7	3.9 ± 0.5	4.5 ± 0.3	5.3 ± 0.3
NAEB	10	13.7 ± 1.0	4.5 ± 0.3	4.1 ± 0.4	5.2 ± 0.5
PVC	9	14.9 ± 1.4	4.8 ± 0.3	4.6 ± 0.6	5.5 ± 0.6
Two causes	10	12.8 ± 0.8	4.5 ± 0.3	3.9 ± 0.3	4.4 ± 0.4
Others	11	13.9 ± 0.8	4.4 ± 0.3	4.2 ± 0.3	5.4 ± 0.3

F value		0.57	0.61	0.44	0.99
*P *value		0.75	0.72	0.85	0.43

Abbreviation: CVA, cough variant asthma; UACS, upper airway cough syndrome; GERD, gastroesophageal reflux disease; NAEB, nonasthmatic eosinophilic bronchitis; PVC, post virus cough

**Table 4:** Comparison on physical and mental component summary of SF-36 between different etiologies (n = 110)

**Causes**	**Cases**	**PCS**	**MCS**
UACS	10	61.9 ± 5.2	58.3 ± 6.2
CVA	54	60.6 ± 4.7	57.8 ± 5.8
GERD	6	62.0 ± 5.3	57.8 ± 5.0
NAEB	10	59.6 ± 4.1	55.9 ± 6.3
PVC	9	60.5 ± 5.0	55.9 ± 5.8
Two causes	10	59.7 ± 6.3	56.4 ± 5.6
Others	11	57.6 ± 5.5	57.1 ± 4.4

F value		0.92	0.44
*P *value		0.48	0.85

Abbreviation: CVA, cough variant asthma; UACS, upper airway cough syndrome; GERD, gastroesophageal reflux disease; NAEB, nonasthmatic eosinophilic bronchitis; PVC, post virus cough; PCS, physical component summary; MCS, mental component summary

### Differences of HRQOL between males and females with chronic cough

When evaluated by LCQ, no significant difference in HRQOL was found between males and females as a whole, despite that the feelings of embarrassment, frustration and disturbance of sleep were more obvious in women than in men (Table 5).

**Table 5:** Gender differences in HRQOL of patients with chronic cough as measure by LCQ (n = 110)

**Adverse events caused by chronic cough**	**Males**	**Females**	**t value**	**P value**
Chest or stomach pains	5.3 ± 1.7	5.5 ± 1.7	-0.32	0.75
Phlegm	4.5 ± 2.3	4.6 ± 2.3	-0.17	0.86
Tiredness	5.2 ± 1.6	4.7 ± 1.8	1.28	0.20
Controlled by cough	3.2 ± 1.7	3.1 ± 1.8	0.44	0.66
Embarrassment	4.9 ± 1.6	3.7 ± 1.6	4.02	0.00
Anxiety	4.5 ± 1.9	4.2 ± 1.6	1.01	0.32
Interference with job/other daily tasks	5.2 ± 1.6	5.1 ± 1.8	0.29	0.78
Interference with overall enjoyment	5.4 ± 1.7	5.4 ± 1.7	0.11	0.92
Cough by exposure of paints or fumes	3.8 ± 2.4	3.9 ± 2.2	-0.22	0.83
Disturbance of sleep	5.2 ± 1.9	4.4 ± 1.9	2.16	0.03
Coughing bouts	3.1 ± 1.3	3.1 ± 1.2	-0.16	0.88
Frustration	5.1 ± 1.7	4.5 ± 1.5	2.02	0.04
Feed up	4.2 ± 1.9	4.2 ± 1.6	0.05	0.96
Hoarse voice	5.5 ± 1.4	5.3 ± 1.6	0.65	0.52
Full of energy	4.8 ± 1.6	4.6 ± 1.8	0.78	0.44
Worry about serious diseases	4.4 ± 1.9	4.3 ± 1.7	0.49	0.63
Concerned the others' feelings about your cough	4.8 ± 2.0	5.4 ± 1.6	-1.70	0.09
Interrupted conversation or telephone calls	5.3 ± 1.6	4.8 ± 1.5	1.68	0.10
Annoying partner, family or friends	5.5 ± 1.9	5.5 ± 1.7	-0.08	0.94

### Effects of specific treatment on HRQOL of patients with chronic cough

In 103 patients who got a definite diagnosis for their cough and received the specific treatments, cough resolved completely in 86 patients. Five patients did not respond to the treatment and 12 patients were lost to follow-up and were therefore excluded for the further analysis of the data. As measured with LCQ, the successful treatment of chronic cough obviously improved HRQOL of patients in the physical, psychological and social domains (Table 6). The time intervals between pre- and post-treatment evaluations were 3.9 ± 1.5 (range 2-13) weeks.

**Table 6:** Changes in HRQOL of patients with chronic cough before and after treatment as measured by LCQ (n = 86)

**Score**	**Before treatment**	**After treatment**	**t value**	***P *value**
total score	14.2 ± 2.7	19.5 ± 1.9	13.7	0.000
Physical domain	4.6 ± 1.0	6.3 ± 0.8	13.5	0.000
Psychological domain	4.3 ± 1.0	6.5 ± 0.7	17.4	0.000
Social domain	5.3 ± 1.2	6.7 ± 0.6	10.7	0.000

### Relationships between LCQ and SF-36 or cough threshold C5

There was a weak but significant correlation between the total score of LCQ and PCS (r = 0.39, *P *< 0.0001) or MCS (r = 0.30, *P *< 0.001) of SF-36 respectively. The median of cough threshold C5 was 3.9 μmol/L (0.49 - 62.5 μmol/L, 95% confidence interval: 8.0-9.3 μmol/L). No significant correlation was found between Log C5 and LCQ (r = 0.134, *P *= 0.253), PCS (r = -0.092, *P *= 0.43) or MCS (r = -0.22, *P *= 0.06) of SF-36.

## Discussion

The quality of life is an important outcome parameter in the study of chronic cough. To investigate HRQOL of Chinese patients with chronic cough, we compared the scores of items in SF-36 between patients with chronic cough and healthy volunteers. The reasons for selection of SF-36 were that SF-36 is one of the most common instruments in general health survey, its reliability and validity have been established with extensive application [16]. Moreover, the measurements by the specific instruments such as LCQ reflect HRQOL in the patients with chronic cough well, but do not accurately represent the health status in healthy volunteers because of lacking cough, which might result in the poor comparability in HRQOL between coughers and non-coughers. Finally, SF-36 has been used for the assessment of the quality of life in the other chronic respiratory diseases such as asthma and chronic obstructive pulmonary disease, and always achieved great success [17,18].

The results showed that the quality of life in patients with chronic cough deteriorated significantly when compared with healthy volunteers. The scores of patients with chronic cough were lower in 7 testing multi-item scales of SF-36 except for physical pain than those of healthy volunteers. Among the affected domains, the decrease in the scores of RE, GH and RP related to emotional problems was more obvious. These observations were in accordance with previous reports on HRQOL in patients with chronic cough [5-8,19].

There was comparable HRQOL among the patients in spite of causes of chronic cough, which confirmed the findings of French and Canonica who had observed that HRQOL in the chronic coughers was unrelated to the causes of cough [8,20]. The impact of cough on quality of life was to a large extent dependent upon the frequency and intensity of cough [5]. Our previous study has also shown that the cough severity was not significantly different among different causes of chronic cough [21]. Although the pathogenesis of chronic cough is associated with the cause, airway inflammation and increased sensory nerve sensitivity in the airway is a common pathway and may play an important role [22,23]. Therefore, it is reasonable that different causes eliciting chough result in the similar cough severity and HRQOL in the cohort of patients.

Gender is one possible factor determining the quality of life of patients with chronic cough. Most studies have demonstrated that women accounted for the majority of patients with chronic cough seeking medical care, with the ratio of 1:1.2-3.6 between males and females [24,25]. It seems that negative HRQOL caused by cough was more conspicuous in women than in men, and might be attributed to the predominance of females in the patients with chronic cough [26]. In contrast, the present study showed that only several adverse events including embarrassment, frustration and sleep disorder were more apparent in women than men as measured with LCQ, and did not confirm the gender difference in overall HRQOL of patients with chronic cough. The selecting bias of patients should not be an explanation since we recruited the patients consecutively. It is likely that in previous studies reporting gender differences in HRQOL, female patients were coughing more frequently than men, and in this study, coughing bouts were identical in men and women.

Another possibility is that the quality of life in the patients with chronic cough visiting hospital was affected in a similar level regardless of gender. Women with chronic cough were more likely to see a doctor than men because of embarrassment and the other psychosocial issues provoked by cough-related urinary incontinence [26]. However, it only explains more females in patients with chronic cough seeking medical advice. When a man went to hospital due to his cough, it meant that his HRQOL was decreased to a level comparable with females, thereby not leading to the significant differences in HRQOL between men and women.

When cough resolved with successful treatment specific for the cause, the quality of life in the patients could be significantly improved as judged by total score and domain scores in LCQ, which is in accordance with the previous study [5]. Therefore, HRQOL assessments of chronic cough, as a precise and quantitative measurement, could be applied in monitoring of cough severity, evaluation of treatment efficacy and verification of new therapeutic regimen.

Birring and Kalpaklioglu have found that there was a negative correlation between LCQ and the other tools such as cough symptom score and visual analogue scale respectively [6,27]. Recently, Kelsal has demonstrated that a correlation existed between LCQ and cough frequency recording [28]. Our study has shown that there was a weak but significant correlation between LCQ and SF-36. The similar relationship was verified between SF-36 and a Dutch version of LCQ [29]. These evidences suggest that LCQ, like the other cough-specific instruments, could accurately measure the HRQOL changes in patients with chronic cough. As indicated by our data, the Chinese version of LCQ is helpful for evaluation of the health status in Chinese patients with chronic cough.

There was no obvious correlation between LCQ and capsaicin cough threshold, which was similar to the results reported by Birring [6] and Chang [30]. It may be due to the different implications of HRQOL and capsaicin cough sensitivity in assessment of cough severity. Capsaicin cough threshold mainly represents the susceptibility of cough while HRQOL reflects the perception of multidimensional damage caused by cough. Therefore, HRQOL and capsaicin cough threshold may measure different aspects of the severity of cough and can be complemented by each other.

## Conclusion

In conclusion, HRQOL is adversely affected by chronic cough but improves when cough resolves. In general, the changes in HRQOL are not related to the causes of cough and gender differences although some negative emotions are more obvious in female Chinese patients. The Chinese version of LCQ is useful tool for evaluation of the health status in Chinese patients with chronic cough.

## Competing interests

The authors declare that they have no competing interests.

## Authors' contributions

WM was in charge of collection of cases and writing the manuscript, LY was in charge of collection, process, and statistical analysis of data and took part in review of the manuscript, YW, XL and HL took part in the collection of cases and review of the manuscript, ZQ was in charge of design and coordination of the program, review and correction of the manuscript. All authors read and approved the final manuscript.

## Supplementary Material

Additional file 1 **LCQ in Chinese**. The Chinese version of Leicester Cough Questionnaire provided is the translation of the original one. Click here for file
