# Supplementary material for: Changes in health-related quality of life and clinical implications in Chinese patients with chronic cough
Source: Cough. 2009 Sep 25;5:7. doi: 10.1186/1745-9974-5-7 (PMC2760496; doi:10.1186/1745-9974-5-7)
Supplement: Additional file 1 — LCQ in Chinese. The Chinese version of Leicester Cough Questionnaire provided is the translation of the original one. [file 1745-9974-5-7-S1.DOC]

**莱塞斯特咳嗽生命质量问卷**

姓名： 性别： 年龄： 病例号：

下列问题是为评估咳嗽对您生命质量的全方位影响而设计的。请认真阅读每一个问题，在您认为最好的答案上画圈。请如实回答所有问题。

1 近两周来，咳嗽会让您胸痛或肚子痛吗？

①一直都会 ②大多数时间会 ③时常会 ④有时会 ⑤很少会 ⑥几乎不会 ⑦一点也不会

2 近两周来，您会因咳嗽有痰而烦恼吗？

①每次都会 ②多数时间会 ③不时会 ④有时会 ⑤偶尔会 ⑥极少会 ⑦从来不会

3 近两周来，咳嗽会让您感到疲倦吗？

①一直都会 ②大多数时间会 ③时常会 ④有时会 ⑤很少会 ⑥几乎不会 ⑦一点也不会

4 近两周来，您觉得能控制咳嗽吗？

①一点也不能 ②几乎不能 ③很少能 ④有时能 ⑤常常能 ⑥多数时间能 ⑦一直都能

5 近两周来，咳嗽会让您觉得尴尬吗？

①一直都会 ②大多数时间会 ③时常会 ④有时会 ⑤很少会 ⑥几乎不会 ⑦一点也不会

6 近两周来，咳嗽会让您焦虑不安吗？

①一直都会 ②大多数时间会 ③时常会 ④有时会 ⑤很少会 ⑥几乎不会 ⑦一点也不会

7 近两周来，咳嗽会影响您的工作或其他日常事务吗？

①一直都会 ②大多数时间会 ③时常会 ④有时会 ⑤很少会 ⑥几乎不会 ⑦一点也不会

8 近两周来，咳嗽会影响您的整个娱乐生活吗？

①一直都会 ②大多数时间会 ③时常会 ④有时会 ⑤很少会 ⑥几乎不会 ⑦一点也不会

9 近两周来，接触油漆油烟会让您咳嗽吗？

①一直都会 ②大多数时间会 ③时常会 ④有时会 ⑤很少会 ⑥几乎不会 ⑦一点也不会

10 近两周来，咳嗽会影响您的睡眠吗？

①一直都会 ②大多数时间会 ③常常会 ④有时会 ⑤很少会 ⑥几乎不会 ⑦一点也不会

11 近两周来，您每天阵发性咳嗽发作多吗？

①持续有 ②次数多 ③时时有 ④有一些 ⑤偶尔有 ⑥极少有 ⑦一点也没有

12 近两周来，您会因咳嗽而情绪低落吗？

①一直都会 ②大多数时间会 ③时常会 ④有时会 ⑤很少会 ⑥几乎不会 ⑦一点也不会

13 近两周来，咳嗽会让您厌烦吗？

①一直都会 ②大多数时间会 ③时常会 ④有时会 ⑤很少会 ⑥几乎不会 ⑦一点也不会

14 近两周来，咳嗽会让您声音嘶哑吗？

①一直都会 ②大多数时间会 ③时常会 ④有时会 ⑤很少会 ⑥几乎不会 ⑦一点也不会

15 近两周来，您会觉得精力充沛吗？

①一点也不会 ②几乎不会 ③很少会 ④有时会 ⑤常常会 ⑥多数时间会 ⑦一直都会

16 近两周来，咳嗽会让您担心有可能得了重病吗？

①一直都会 ②大多数时间会 ③时常会 ④有时会 ⑤很少会 ⑥几乎不会 ⑦一点也不会

17 近两周来，咳嗽会让您担心别人觉得您身体不对劲吗？

①一直都会 ②大多数时间会 ③时常会 ④有时会 ⑤很少会 ⑥几乎不会 ⑦一点也不会

18 近两周来，您会因咳嗽中断谈话或接听电话吗？

①每次都会 ②大多数时间会 ③时常会 ④有时会 ⑤很少会 ⑥几乎不会 ⑦一点也不会

19 近两周来，您会觉得咳嗽惹恼了同伴﹑家人或朋友？

①每次都会 ②多数时间会 ③不时会 ④有时会 ⑤偶尔会 ⑥极少会 ⑦从来不会

感谢您的参与！

签 名：

填表日期：

调查医生：

注：莱塞斯特咳嗽生命质量问卷评分方法

1. 区域（问题） 评定分数

① 生理：包括问题1，2，3，9，10，11，14，15 生理=（ ）÷8=

② 心理：包括问题4，5，6，12，13，16，17 心理=（ ）÷7=

③ 社会：包括问题7，8，18，19 社会=（ ）÷4=

1. 区域得分=区域各项问题总分÷问题数（分值1-7） 总分=
2. 总分=三区域得分之和（分值3-21）

本表经Birring教授授权从Leicester Cough Questionnaire (LCQ)翻译而来，原文见*Thorax,* 2003, 58: 339-343.
